# Ex vivo evaluation of a novel through-the-scope traction device for endoscopic submucosal dissection

**DOI:** 10.1055/a-2576-5837

**Published:** 2025-06-17

**Authors:** Ludovico Alfarone, Roberto De Sire, Boris Rosenbaum, Ali Aidibi, Christophe Cellier, Cesare Hassan, Alessandro Repici, Gabriel Rahmi, Roberta Maselli

**Affiliations:** 1 551905 Endoscopy Unit, IRCCS Humanitas Research Hospital Department of Gastroenterology, Rozzano, Italy; 2 9307 Gastroenterology, IBD Unit, Department of Clinical Medicine and Surgery, Università degli Studi di Napoli Federico II, Napoli, Italy; 3 55647 Gastroenterology and Endoscopy, Hôpital Européen Georges Pompidou, Paris, France; 4 437807 Department of Biomedical Sciences, Humanitas University, Pieve Emanuele, Italy; 5 437807 Department of Biomedical Sciences, Humanitas University, Pieve Emanuele, Milan, Italy

**Keywords:** Endoscopy Upper GI Tract, Endoscopic resection (ESD, EMRc, ...), Quality and logistical aspects, Performance and complications, Training

## Abstract

**Background and study aims:**

Endoscopic submucosal dissection (ESD) is a technique used for resection of large neoplastic lesions, providing great oncological outcomes. However, ESD is technically challenging with a long learning curve, high complication rates, and extended procedure times. To address these limitations, various traction-assisted methods have been developed. This study evaluated a novel through-the-scope traction device (TRACMOTION, Fujifilm, Japan), which offers consistent and adjustable traction, in a pilot multicenter randomized ex vivo trial.

**Patients and methods:**

The study included six endoscopists with limited ESD experience performing ESD on ex vivo porcine stomach models. Participants were randomized into two groups: those using TRACMOTION-assisted ESD (TM-ESD) and those performing conventional ESD (C-ESD). Each trainee completed six procedures, split equally between both groups. The primary outcome was resection speed, whereas secondary outcomes included en bloc, R0 resection rates, perforation rates, and rates of speedy procedures (>20 mm²/min).

**Results:**

The TM-ESD group achieved a higher median resection speed of 20.75 mm²/min (95% confidence interval [CI] 14.56–29.25) in comparison to 15.10 mm²/min (95% CI 12.8–16.6) in the C-ESD group ( *P* =0.02). Perforation rates were significantly lower in the TM-ESD group (11.1% (95% CI 0.01–0.34) vs. 61.1% (95% CI 0.35–0.82; *P* =0.0002). No significant differences were observed in en bloc or R0 resection rates between the groups.

**Conclusions:**

TRACMOTION significantly improves the efficiency of ESD, reducing procedure time and complication rates. This traction device could potentially facilitate broader adoption of ESD in clinical practice. However, further research in human trials is necessary to validate these findings.

## Introduction

Endoscopic submucosal dissection (ESD) has emerged as a revolutionary technique for en bloc resection of large early-stage neoplastic lesions in the gastrointestinal tract, providing an effective alternative to traditional surgical methods. This technique offers significant advantages, such as reduced invasiveness and preservation of organ function 1 .

ESD has shown its superiority in terms of oncological outcomes over other endoscopic resection techniques, particularly in comparison with endoscopic mucosal resection (EMR). However, ESD remains technically challenging compared with more conventional techniques, including polypectomy and EMR, resulting in higher complication rates and longer operative times 2 . Furthermore, the ESD learning curve for beginners is long. The spread of this technique in the Western world is thus limited, despite the organization of specific training courses and recommendations published by the European Society of Gastrointestinal Endoscopy in the form of a curriculum 3 4 . ESD is complex because of the inherent in difficulty in accessing the submucosal space and achieving traction adequate for dissection of the lesion from the underlying muscular tissue, while maintaining good scope maneuverability during the procedure 5 . To address these challenges, various techniques have been developed to enhance the precision and safety of ESD. Among them, traction-assisted ESD methods have gained considerable attention. By facilitating better submucosal exposure and access, traction-assisted methods can indeed potentially reduce procedure time and complications 6 7 8 .

However, traditional traction devices, such as clip-and-line and double-clip and rubber band systems, typically provide a non-adaptive traction force that can diminish as dissection progresses, limiting their effectiveness for large tumors. To overcome this issue, a new tool providing an adaptive traction force has been developed, with great preliminary results 9 10 . Nevertheless, both traditional and novel traction methods require switching between multiple devices, increasing overall procedure time and making the process cumbersome.

Consequently, there is a pressing need for advanced traction-assisting devices that offer consistent and adjustable traction throughout the entire ESD procedure. Such innovations would enhance continuous and optimal submucosal exposure, thereby improving the efficacy and safety of the technique.

In this context, a novel through-the-scope traction device equipped with 360° rotatable jaws (TRACMOTION, Fujifilm, Japan) was designed for easy grasping and re-grasping of large lesions, enabling consistent and adjustable tension, and facilitating a more controlled and efficient dissection process.

The aim of this multicenter, randomized, ex vivo animal trial was to compare overall outcomes between TRACMOTION-assisted ESD (TM-ESD) and conventional ESD (C-ESD) in trainees with limited ESD experience.

## Methods

### Study design and outcomes

This was a pilot, randomized, controlled, and prospective ex vivo animal trial among six endoscopists at two labs (Humanitas Research center and University, Milan, Italy; Biosurgical Research Laboratory of the Fondation Alain Carpentier, Paris, France). All enrolled endoscopists had more than 2 years of experience in endoscopy and had performed more than 500 gastroscopy procedures. All the endoscopists were considered trainees in ESD, having never performed a full case in humans and done five to 20 ESD cases in models. None of the trainees had prior exposure to the traction device. At the beginning of the study, participants attended a structured learning session with instructions on how to set up and use the traction device.

They were enrolled to perform ESD in porcine stomach ex vivo model with or without use of the TRACMOTION device, as follow. Trainees in the ESD in the TM-ESD group used TRACMOTION and the standard device (Flushknife BTs). Trainees in ESD in the C-ESD group using Flushknife (standard technique).

Each beginner was asked to perform six ESDs, three for each group. Beginners were randomly (manual randomization) assigned to do the first three TM-ESDs and then three C-ESDs, or vice versa.

The primary outcome of the study was a comparison of resection speed between TM-ESD and C-ESD. Secondary outcomes were en bloc, R0 resection, and perforation rates, as well as the rate of speedy procedures (>20 mm²/min). General sentiments from the endoscopists about traction device performance also was assessed.

### Traction device evaluation

Performance of the TRACMOTION device was assessed using a 5-point Likert scale, where 0 = very bad, 1 = bad, 2 = acceptable, 3 = good, and 4 = very good. Endoscopists evaluated the device based on five specific criteria: 1) ease of device delivery; 2) ability to grasp tissue; 3) traction effectiveness; 4) interference with the ESD procedure; and 5) overall satisfaction. Each operator was informed in advance about the evaluation criteria to ensure standardized assessment. Ratings were recorded immediately after each procedure to minimize recall bias.

### Porcine stomach ex vivo model

Pigs were obtained from the Experimental Animal Center of Humanitas University, Milan, Italy and Biosurgical Research Laboratory of the Fondation Alain Carpentier, Paris, France. The animal protocol was developed to minimize pain to the pigs. The isolated pig stomachs were obtained from pigs that were euthanized after the end of other experimental projects. The experimental protocol was approved by the laboratory animal care committee and was in accordance with the ethical standards for experimental animal labs. All animal experiments complied with the ARRIVE guidelines and were carried out in accordance with the National Institutes of Health Guide for the Care and Use of Laboratory Animals (Eighth edition, 2011).

### Procedure

Isolated pig stomachs were obtained and about 15 cm of the esophagus and duodenal stump were maintained. The duodenal stump was clamped with forceps, and then a gastroscope was inserted from the esophageal stump to inflate and observe the airtightness of the stomach and the integrity of the mucosa. Participants were instructed to perform gastric en bloc ESDs. Preparation before ESD was standardized as follows: 1) comparable lesion size was confirmed visually while by applying current to the open snare (Fujifilm, Medwork, Resection Master, 30 mm); and 2) location in the pig stomach was similar for each ESD (i.e., in the proximal stomach approximately 10 cm distal to the cardia greater curvature.

### C-ESD technique

To avoid laboratory-related biases we used the same equipment in both laboratries: Fujifilm Flushknife BTs 3 mm, injecting needle (Manta, Fujifilm), injecting solution consisting of saline mixed with methylene blue or indigo carmine, and ERBE VIO3 electrosurgical generator. For the C-ESD group, a standard gastroscope (Fujifilm Endoscopy, Tokyo, Japan) was used with a short transparent cap attached to the tip of the endoscope.

First, the endoscope was placed in the desired position. After marking, the lesion was lifted by submucosal injection. Then, circumferential incision and dissection of the submucosa were performed with the aim of en bloc resection of the lesion. After, the resected specimen was retrieved to measure its size and assess completeness of resection (en bloc, R0).

### TM-ESD technique with traction device

The TRACMOTION device improves and enhances the submucosal dissection phase through its ability to grasp, tract, and rotate tissue.Video 1

For the TM-ESD group, a double-channel upper endoscope (EI 740-D/S, Fujifilm) with 3.7-mm instrument channels was used for all procedures.

**Fig. 1:**
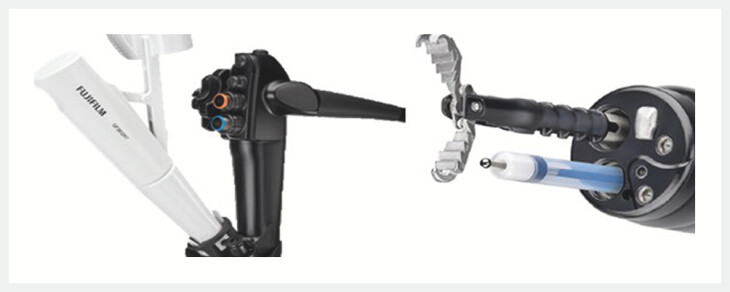
The through-the-scope traction device composed of an actuating distal end interconnected to a hand controller mounted on the endoscope.

A single-operator, through-the-scope, articulating traction device (TRACMOTION, Fujifilm Endoscopy, Tokyo, Japan) was designed for TM-ESD procedures. It is composed of two interconnected parts: an actuating distal end and a hand controller mounted on the endoscope. On the distal end is a grasper that can be rotated and articulated ( Fig. 1 ).

**Fig. 2:**
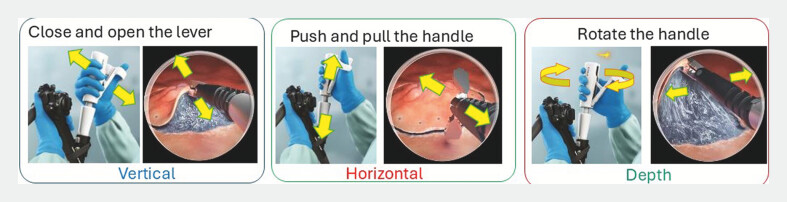
Range of actions with TRACMOTION to gain required traction.

**Fig. 3:**
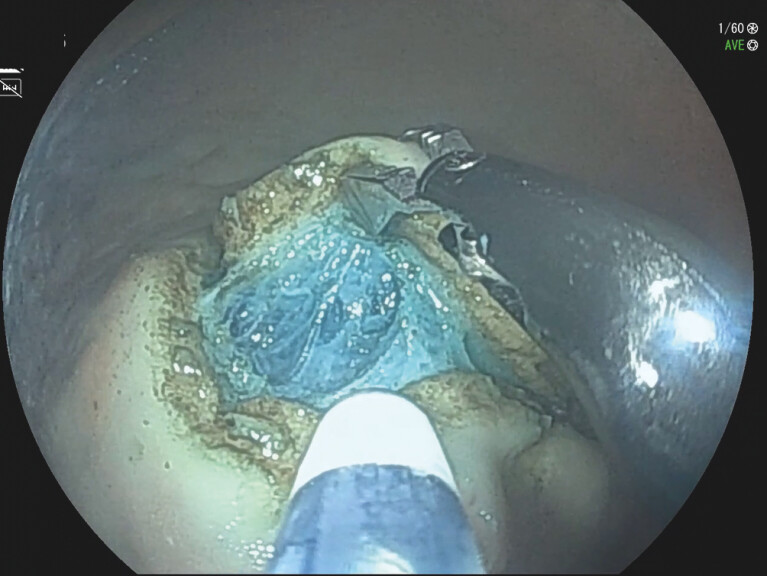
Video image.

The device has five degrees of freedom thanks to its combined articulation and rotational capabilities, which enable extension and flexion, clockwise and counterclockwise rotation, advancement, and retraction. The 3.7-mm instrument channels, which are equivalent to the larger channel of a double-channel endoscope, are compatible with the device. Using an adapter, the hand controller is fixed to the biopsy port. The thumb handle and the distal pivotable shaft are the functional components of the controller. The right hand of the endoscopist is used to operate the controller, mirroring the movements of the distal end. Grasping, locking, and flexion are accomplished by gripping the thumb handle, whereas rotation and advancement are accomplished by analogous movements of the pilotable shaft ( Fig. 2 , Fig. 3 ) ( Video 1 ).

### Data collection and definitions

En bloc resection was defined as resection of the target lesion in one piece. Endoscopic R0 resection was diagnosed if lateral resection margins appeared outside of markings. Specimen surface area was calculated as: area (mm ^2^ ) = (smaller diameter (mm)/2) × (larger diameter (mm)/2) × π. Procedure duration (min) was calculated as time between the first submucosal injection and dissection of the last submucosal fiber. Resection speed (mm ^2^ /min) was calculated as specimen area divided by procedure duration. Speedy procedures were defined as having resection speed >20 mm²/min. Perforation was defined as a muscular defect occurring during the ESD procedure.

CADEYE processor (Fujifilm) was used to record all procedures; videos were revised for data collection. Data were collected in a specific case report form to create a database with data from the two labs. Data were analyzed by a statistician blinded to groups and labs

### Statistical analysis

Continuous variable distribution was assessed using the Shapiro-Wilk test of normality and reported as a mean with standard deviation (SD) or a median with interquartile range (IQR). Categorical variables were expressed as counts and percentages. Associations between continuous variables were assessed using the Kruskal-Wallis test, and those between categorical variables were assessed using a χ ^2^ test or Fisher’s exact test as appropriate. A two-sided *P* <0.05 was considered to indicate statistical significance. All statistical analyses were performed with STATA (ver. 18, Texas, United States). All authors had access to the anonymized study data and approved the final manuscript

## Results

### Baseline characteristics

Overall, 36 gastric ESD procedures (18 in the TM-ESD group and 18 in the C-ESD group) in 12 ex vivo pig stomachs were performed by six endoscopists (four at the Italian lab and two at the French lab) over 3 days (two at the Italian lab, one at the French lab)

Mean lesion size was 37.5 mm (SD 6.41; 95% CI 32.53–42.47) and 36.7 mm (SD 7.67; 95% confidence interval [CI] 31.73–41.67) in the TM-ESD and C-ESD groups respectively, with no significant difference ( *P* =0.44).

### Outcomes

#### Speed

**Fig. 4:**
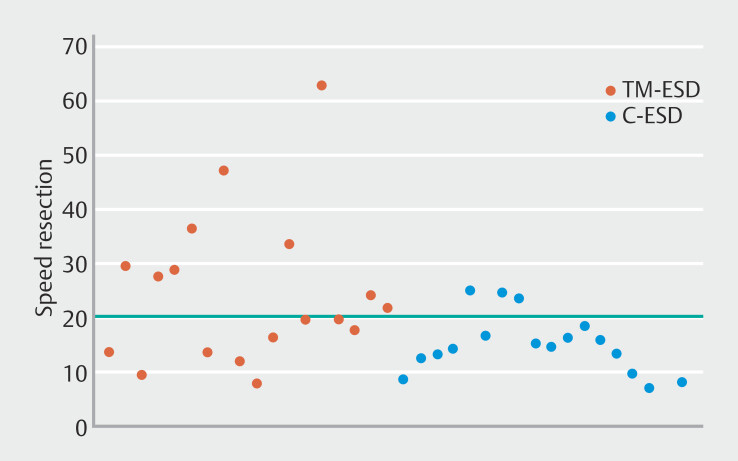
Rate of speedy procedures.

The TM-ESD group achieved a significantly higher median resection speed of 20.75 mm²/min (95% CI 14.56–29.25) in comparison to 15.10 mm²/min (95% C I 12.8–16.6) in the C-ESD group ( *P* =0.02). A significantly higher rate of speedy procedures (resection speed >20 mm²/min) was reported in the TM-ESD group in comparison with the C-ESD group (50% (95% CI 0.260–0.739) vs. 16.7% ((95% CI 0.0358–0.4142) *P* = 0.03), leading to mean procedure times of 41.3 minutes (SD 24.28; %95 CI 36.33–46.27) and 57.1 minutes (SD 23.7; 95% CI 52.23–62.17), respectively ( *P* =0.03) ( Fig. 4 ). Median dissection phase speed was significantly higher in the TM-ESD group than in the C-ESD group (32.30 mm²/min 95% CI 24.61- 67.68 vs 21.14 mm²/min 95% CI 18.62–29.62; *P* =0.01), whereas no difference in terms of median incision phase speed was reported between the two groups (58.57 mm²/min 95% CI 49.47–70.95 and 52.40 mm²/min 95% CI 47.51–69.62 in the TM-ESD and C-ESD groups, respectively; *P* =0.54)

The rate of ESDs completed within 1 hour was comparable between the two groups 88.9% (95% CI 0.65–0.98) for TM-ESD vs. 72.2% (95% CI 0.46–0.90) for C-ESD; *P* =0.2).

#### En bloc and R0 resection

**Fig. 5:**
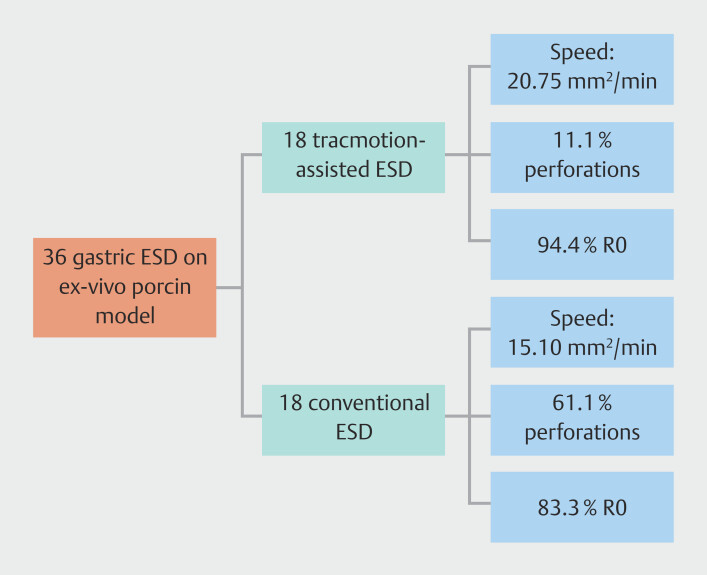
Study design and main outcomes

No significant differences were found in terms of en bloc resection rate (100%; 95% CI 0.81–1.00) for TM-ESD vs. 88.9% ;95% CI 0.65–0.98 for C-ESD; *P* =0.5) and R0 resection rate (94.4%; CI 0.72–0.99 for TM-ESD vs. 83.3%; 95% CI 0.58–0.96) for C-ESD; *P* =0.3) ( Fig. 5 ).

#### Perforation

The TM-ESD group had a significantly lower perforation rate compared with the C-ESD group (11.1%; 95% CI 0.01–0.34 vs. 61.1%; 95% CI 0.35–0.82) ( *P* = 0.0002) ( Table 1 ).

**Table:** **Table 1** Main procedure outcomes.

Outcomes	TRACMOTION	Conventional	*P* value
Median speed resection	20.75 mm²/min	15.10 mm²/min	0.02
En bloc resection	18/18 (100%)	16/18 (88.9%)	0.5
R0 resection	17/18 (94.4%)	15/18 (83.3%)	0.3
Perforation	2/18 (11.1%)	11/18 (61.1%)	0.0002
Completed procedure in 1 hour	16/18 (88.9%)	13/18 (72.2%)	0.2

#### Device performance

Regarding TRACMOTION performance, the mean score for device delivery was 3.66 (SD 0.59 95% CI –1.31–8.63). In terms of grasping tissue capability, a score of 4 was reported for every procedure. The mean score was 3.83 (SD 0.38 95% CI –1.14–8.80) for traction capability. Interference with the ESD procedure was rated with a mean score of 3.44 (SD 0.61 95% CI –1.53–8.41). Finally, regarding overall satisfaction with TRACMOTION, the mean score was 3.61 (SD 0.60, 95% CI –1.36 to 8.58) ( Table 2 ).

**Table:** **Table 2** TRACMOTION performance assessed by endoscopist through a 5-point Likert scale.

TM-ESD procedures	Device delivery (0–4)	Grasping tissue (0–4)	Traction (0–4)	Interference with ESD (0–4)	Total satisfaction (0–4)
N 1	4	4	4	4	4
N 2	4	4	4	4	4
N 3	4	4	3	4	3
N 4	4	4	3	4	4
N 5	4	4	4	4	4
N 6	4	4	4	4	4
N 7	3	4	4	3	3
N 8	4	4	4	4	4
N 9	3	4	4	3	4
N 10	2	4	3	2	2
N 11	3	4	4	3	3
N 12	4	4	4	4	4
N 13	4	4	4	3	4
N 14	4	4	4	3	4
N 15	4	4	4	3	3
N 16	3	4	4	3	3
N 17	4	4	4	3	4
N 18	4	4	4	4	4
ESD, endoscopic submucosal dissection; TM-ESD, TRACMOTION-assisted endoscopic submucosal dissection.					

## Discussion

Our study shows that TRACMOTION could be of significant assistance to beginners performing ESD procedures, decreasing both procedure time and perforation rate.

Our findings are relevant for several reasons. First, high procedure time is one of the main factors that have undermined adoption of and learning about ESD in the West. In fact, a lengthy procedure not only worsens patient outcomes but also undermines the ability of the health care system to be able to respond adequately to the needs of all patients. As a matter of fact, our study found that TRACMOTION significantly increased speed, allowing attainment of dissection speeds better than those reported in previous studies of gastric ESD performed by trainees 11 .

TRACMOTION offers a significant advantage during the submucosal dissection phase due to its ability to provide continuous and adjustable traction, facilitating better exposure and lesion control. These characteristics enable more efficient and safer dissection. Importantly, our findings suggest that TRACMOTION does not significantly compromise the speed of the mucosal incision phase. This indicates that switching instruments between the incision and dissection phases may not be mandatory, potentially streamlining the procedure and reducing procedure complexity.

Second, perforation is arguably the most serious event for an endoscopist and can lead to life-threatening situations. Although many efforts have been made in recent years to limit this risk, the perforation rate remains high, given the technical difficulty of this procedure 2 .

In this study, perforations occurred significantly less frequently in the TRACMOTION group. If we consider the overall number of procedures and the study design, the difference is huge (11% vs 61%) and consistent with that reported by a previous study on the same device 12 ; this last-mentioned study reported lower perforation rates in both arms in comparison to our study, especially in the conventional group. Nevertheless, our trainees had less experience in ESD. Moreover, the high perforation rate appears consistent with those reported by a previous study of beginners performing gastric ESD 13 .

These results underscore why perforation is the biggest concern for beginners approaching ESD, and further suggest that TRACMOTION may be helpful in overcoming this problem by allowing the procedure to be completed by beginners with a lower risk of perforation.

Third, the previously mentioned factors also undermine the potential for training. In fact, the excessive length of the procedure and constant fear of perforation reduce the amount of time devoted to training and the number of ESD steps that beginners may be able to practice. Therefore, TRACMOTION could improve the learning curve of trainees, allowing them to easily achieve submucosal space exposure and traction and, thus, complete ESD procedures with less fear of perforation and in an acceptable operative time.

Furthermore, TRACMOTION differs from other more widely used traction devices because it does not require any device exchanges or a cumbersome process to secure it. What is more, it allows the force and orientation of traction to be changed dynamically during the procedure, ensuring continuous and sustained traction throughout the ESD 6 9 . These features were highly appreciated, as evidenced by the high satisfaction scores given by the endoscopists in our study, which reflect ease and reliability in introducing and positioning the device and effectiveness in securely holding the tissue and providing stable and adjustable traction throughout the procedure.

Because the device is fixed, one of the most common criticisms has been the possibility of interference with the ESD procedure. However, TRACMOTION was not reported to interfere with ESD procedures by endoscopists involved in our study. An additional advantage of TRACMOTION is the atraumatic nature of its graspers, which allow for safe and effective tissue manipulation. This design enables repeated grasping and re-grasping without causing mucosal damage, ensuring stable and controlled traction throughout dissection. Furthermore, the device contributes to endoscope stabilization, which enhances precision during submucosal dissection.

This study has some strengths. The greatest one is its prospective, randomized, controlled, multicenter design, which allowed us to obtain reliable results while minimizing risk of bias. Moreover, the exclusive enrollment of beginners decreased the potential impact of different level of expertise and skills in ESD procedures.

Our study also has limitations. The ex vivo animal model hindered us from drawing definitive conclusions about use of TRACMOTION in humans. In particular, risk of intraprocedural bleeding and the capability to handle it have not been assessed in this study and may represent relevant drawbacks of this device. However, risk of intraprocedural bleeding and its treatment are not considered factors that significantly limit the widespread adoption of ESD or impair learning of the technique, unlike the lengthy procedure time and risk of perforation. Second, we lack a comparison with other traction devices, such as clip-and-line and double-clip and rubber band traction, hindering us from establishing which traction device may be better. Future comparative studies with other countertraction techniques are eagerly awaited. The main aim of the study was not to merely compare procedure outcomes of different traction devices but to provide a pilot randomized controlled trial assessing the benefit of TRACMOTION for beginners performing ESD. Third, the small sample size could undermine reproducibility of our findings and it hindered us from carrying out multivariate analysis to detect risk factors significantly associated with efficacy and safety outcome. Fourth, our study assessed performance of the TRACMOTION device only in a proximal gastric site, limiting generalizability of our findings. We can speculate, however, that the features of TRACMOTION device and the stabilizing effect of graspers could improve endoscope stability, making submucosal dissection easier n anatomically challenging locations, such as the lesser curvature or anterior wall of the stomach. Future studies should explore TRACMOTION performance in these locations.

Finally, although TRACMOTION demonstrated significant advantages in terms of resection speed and reduced perforation rates, its widespread adoption may be limited by certain practical concerns. First, the need for a double-channel endoscope presents a challenge, because these endoscopes are less commonly available, more expensive, and often have lower resolution compared with standard single-channel gastroscopes. This may restrict use of TRACMOTION to specific centers with access to such equipment. In addition, cost considerations related to both the device itself and the required endoscope must be taken into account, particularly in settings with budget constraints. Moreover, applicability of TRACMOTION may be limited to lesions that are accessible with a gastroscope, potentially excluding more challenging anatomical locations. However, the promising results reported in our study may provide reassurance that designing future larger multicenter comparative studies is warranted.

## Conclusions

In summary, TRACMOTION appears to be a useful ESD traction device that may allow beginners to perform ESD faster and more safely. In addition, such a device could facilitate the learning curve and lead to democratization and widespread adoption of the technique. These promising preliminary results need to be confirmed by further studies, especially in humans.
